# Transportation Insecurity, Social Support, and Adherence to Cancer Screening

**DOI:** 10.1001/jamanetworkopen.2024.57336

**Published:** 2025-01-30

**Authors:** Alexa L. Pohl, Aderinsola A. Aderonmu, Joshua D. Grab, Leora A. Cohen-Tigor, Arden M. Morris

**Affiliations:** 1 S-SPIRE Center, Department of Surgery, Stanford University School of Medicine, Stanford, California

## Abstract

**Importance:**

Transportation insecurity and lack of social support are 2 understudied social determinants of health that contribute to excess morbidity, mortality, and acute health care utilization. However, whether and how these social determinants of health are associated with cancer screening has not been determined and has implications for preventive care.

**Objective:**

To determine whether transportation insecurity or social support are associated with screening adherence for colorectal, breast, and cervical cancer.

**Design, Setting, and Participants:**

This cohort study used data from the publicly available 2018 in-person National Health Interview Survey (NHIS) comprising a noninstitutionalized, civilian adult population of the United States. Participants included adults eligible for colorectal, breast, or cervical cancer screening who participated in the in-depth NHIS interview (1 selected per household). Data were acquired in December 2023 and analyzed through July 31, 2024.

**Exposures:**

Transportation insecurity, represented dichotomously as adults who reported that they have or have not delayed medical care in the past year due to transportation difficulties, and neighborhood social support, represented as factor scores derived from 4 Likert-type questions.

**Main Outcomes and Measures:**

The primary outcome was adherence to the US Preventive Services Task Force screening recommendations in place during 2018 for colorectal, breast, and cervical cancer.

**Results:**

In 2018, of 25 417 NHIS respondents (55% female), 660 (3%) reported delaying medical care because they did not have transportation. In fully adjusted models, transportation insecurity was associated with adherence to breast cancer screening (odds ratio [OR], 0.59 [95% CI, 0.40-0.86]) but not to colorectal (OR, 0.87 [95% CI, 0.65-1.15]) or cervical (OR, 0.73 [95% CI, 0.46-1.13) cancer screening. Social support was associated with colorectal (OR, 1.12 [95% CI, 1.06-1.17]) and breast (OR, 1.13 [95% CI, 1.05-1.22]) cancer screening but not with cervical cancer screening (OR, 1.01 [95% CI, 0.93-1.10]). There were no significant interactions between transportation insecurity and social support for any cancer screening.

**Conclusions and Relevance:**

The presence of transportation insecurity was associated with a 41% reduction in the odds of breast cancer screening. Clinicians should consider screening for transportation needs at the time of mammography referral, as patients may be eligible for programs that can assist with medical transportation needs.

## Key Points

**Question:**

Is transportation insecurity associated with cancer screening rates and, if so, does social support modify this association?

**Findings:**

In a cohort study of 25 417 US adults eligible for colorectal (10 736 adults), breast (5823 adults), and cervical (7932 adults) cancer screening, transportation insecurity was negatively associated with uptake of breast cancer screening, and social support was positively associated with uptake of colorectal and breast cancer screening. There was no evidence of an interaction between social support and transportation insecurity for cancer screening.

**Meaning:**

Findings from this cohort study suggest that transportation insecurity and social support are 2 social determinants of health that may be directly associated with cancer screening rates.

## Teaser

This cohort study uses National Health Interview Survey data to assess whether 2 social determinants of health—transportation insecurity and social support—are associated with screening adherence for colorectal, breast, and cervical cancer among US adults.

## Introduction

Nearly 6 million people in the United States delay or forego medical care annually due to transportation difficulties.^1^ The influence of transportation insecurity, defined as the inability to get from place to place in a safe, predictable, and timely manner,^2^ on preventative care is relatively unknown, although associations have been shown with unnecessary hospitalization, emergency department visits, and excess cancer-related, cardiovascular, and overall mortality.^3,4,5^ Some of the correlates of transportation insecurity are common sense (eg, income, education, and car ownership), but others may be surprising; for example, urban and suburban residents are more likely to report transportation insecurity than rural residents.^6^ Although the availability of the Geographic Information Systems has provided insight into the geographic accessibility of health care measured in miles or minutes, it conflates the proximity of resources with the ability of patients to access them. In fact, 15% of American households are car deficient, with another 7% lacking a vehicle altogether, meaning that 22% of Americans’ travel decisions include factors beyond how far they are willing to drive for health care.^7,8^ Indeed, when considering the association between travel distance and cancer screening, the heterogeneity,^9,10^ nonlinear effects, and interactions with vehicle access and deprivation reported in the literature^11,12^ suggest distance is only one of many transportation-related factors directing health care behaviors. As manifestations of transportation insecurity include lateness, skipping trips, social isolation, worry about transportation, and excessive time spent planning, waiting, and traveling,^2^ a more robust system is needed to capture how these aspects affect health care, including cancer screening.

One strategy to cope with transportation insecurity, relying on others for a ride, is based on potentially compensatory social support.^13,14^ Social support, defined as the prosocial exchange of tangible and intangible resources between at least 2 people,^15^ can be broken into subtypes based on the resources exchanged. Material support involves exchange of commodifiable goods and services; emotional support involves providing prosocial intangibles such as affirmation, love, and acceptance; and informational support involves provision of knowledge intended to help the recipient.^16^ Social support has beneficial effects on health by buffering both tangible and intangible stressors and through direct positive effects.^17^ Social networks are vested in neighborhoods, and neighborhood-level social resources may be stronger predictors of health outcomes than local access to health care.^18^ This is especially relevant for those living in poverty, as social exchange networks are used daily to meet needs, including health care.^19^ Qualitative research demonstrates that persons with transportation insecurity leverage informal social support to meet their health-related transportation needs,^13,14^ consistent with the buffering hypothesis. However, whether social support can buffer adverse health outcomes resulting from transportation insecurity remains unknown.

In a large, nationally representative sample, our objectives were to examine (1) the association between transportation security and adherence to recommended cancer screening, (2) the association between social support and adherence to recommended cancer screening, and (3) the possibility of an interaction between transportation security and social support on cancer screening to determine whether a buffer effect could be present.

## Methods

### Study Population

This cohort study used data from the National Health Interview Survey (NHIS), a yearly cross-sectional survey of US residents administered by the National Center for Health Statistics.^20^ Staff conduct technology-augmented interviews at households within predefined sampling regions; adults and children living in institutions are excluded. The survey contains a set of annual questions as well as rotating questions. The 2018 survey featured items on cancer screening, transportation insecurity, and neighborhood social support among its rotating items. Poststratification ensures that the sample population is representative of the noninstitutionalized, nonmilitary adult population of the US. In 2018, the survey had a 64.2% response rate, resulting in 25 417 adults (1 per household) who answered detailed questions about their health. We restricted our analyses to adults eligible for colorectal (50-74 years of age), breast (women 50-74 years of age), or cervical (women 21-65 years of age without prior hysterectomy) cancer screening according to US Preventive Services Task Force (USPSTF) guidelines in effect during 2018. Data were acquired in December 2023 and analyzed through July 31, 2024. This analysis of a deidentified, publicly available dataset was exempt from Stanford University Institutional Review Board oversight. We followed the Strengthening the Reporting of Observational Studies in Epidemiology (STROBE) reporting guideline.

### Outcome

The outcome was adherence to USPSTF cancer screening recommendations at the time of the survey, which we derived from questions regarding whether patients had undergone specific screening tests (eg, colonoscopy) and the interval since the test was completed. For breast cancer screening, participants were deemed adherent if they reported undergoing a mammogram within the past 2 years. For cervical cancer, participants were deemed adherent if they reported having a Papanicolaou test within the past 3 years (ages 21-29 years) or within the past 5 years (ages 30-65 years), assuming that the Papanicolaou test done after age 30 years included human papillomavirus cotesting. For colorectal cancer, participants were deemed adherent if they reported undergoing a fecal immunochemical test within the past year, stool-based DNA test within the past 2 years, flexible sigmoidoscopy within the past 5 years, computed tomography colonography within the past 5 years, or colonoscopy within the past 10 years.

### Exposures

Transportation insecurity was measured as a binary variable in response to the question, “Have you delayed getting [medical] care for any of the following reasons in the past twelve months?” with the answer, “You didn’t have transportation.” Missing cases were effectively deleted listwise while retaining appropriate poststratification weighting of the entire sample (eTable 1 in Supplement 1).

The survey included 4 Likert questions about neighborhood social support. Because the statements potentially encompassed material social support (“People in this neighborhood help each other out,” “There are people I can count on in this neighborhood”), and emotional social support (“People in this neighborhood can be trusted,” “This is a close-knit neighborhood”), we conducted a factor analysis to determine whether these statements represented 1 or more constructs.

### Covariates

Sociodemographic characteristics previously associated with transportation insecurity,^6^ social support,^21^ and cancer screening behavior^22^ included age, gender, race and ethnicity, marital status, employment, income, and educational attainment. We included person-specific age, gender, race and ethnicity, marital status, and employment; personal history of breast, colorectal, or cervical cancer; and individual (ie, not aggregate) household highest educational attainment and income (normalized as federal poverty ratio) as covariates in all models. The NHIS race and ethnicity categories included were Hispanic, non-Hispanic Asian, non-Hispanic Black, non-Hispanic White, and non-Hispanic other (which included 78% American Indian or Alaska Native, 2% multiple race, no primary race selected, and 20% primary race not releasable). Missingness of covariates was less than 1%, with no apparent differences between exposures; the exception was of federal poverty ratio, which was missing for 16% of respondents (eTable 1 in Supplement 1). Therefore, we used the multiple imputation files provided by the National Center for Health Statistics for federal poverty ratio, as recommended in the documentation; all other missing values were effectively handled with listwise deletion.

### Statistical Analysis

To simplify the 4 questions regarding neighborhood social support, we conducted an exploratory factor analysis using the psych package in R. We used a scree plot with observed and simulated eigenvalues to determine the optimal number of factors. After listwise deletion of cases with missing values for any of the 4 social support items, we calculated a polychoric correlation matrix and used the minimum residual method to find loadings for a 1-factor solution. We used bootstrapping (1000 iterations) to find CIs around the factor loadings. We extracted factor scores using the regression solution; no rotation was indicated as this was a single-factor solution. Finally, we calculated indeterminacy indices and the Grice validity coefficient to evaluate the extracted factor scores.

We used frequency tables to describe the population exposed to transportation insecurity or social support and the reference population. The association between transportation insecurity and adherence to recommended cancer screening was first tested in a series of univariable logistic regression models, and then multivariable logistic regression models were used to adjust for confounders. The association between social support and cancer screening adherence was similarly tested with both univariable and multivariable logistic regression models, and bootstrapped (1000 iterations). Covariates remained the same between models, with the exception that gender was included for the colorectal cancer screening model and that only personal history of the relevant cancer was included. Finally, fully adjusted generalized linear models with an interaction term for transportation insecurity and social support were run to test for the possibility of an interaction between these variables.

Survey poststratification weights were maintained throughout the analysis using the survey package in R; sample sizes represent raw data, but all other descriptive and inferential statistics presented use weighting. All multivariable models were conducted with the multiple imputation files for federal poverty ratio provided by the NHIS, accessed through the mitools package. R, version 4.3.1 (R Project for Statistical Computing) was used for analysis. Statistical significance was defined as a 95% CI excluding 1.

## Results

In 2018, over 99% (n = 25 163) of 25 417 NHIS respondents responded to the question about delaying medical care for the assessed reasons, and over 98% (n = 24 909) responded to all 4 Likert questions about neighborhood social support (eTable 1 in Supplement 1). In total, 690 respondents (3%; 63% female and 37% male; 14% Hispanic, 3% non-Hispanic Asian, 24% non-Hispanic Black, 55% non-Hispanic White, and 3% non-Hispanic other, which included 78% American Indian or Alaska Native, 2% multiple race, no primary race selected, and 20% primary race not releasable) reported delaying medical care because they did not have transportation. Respondents who delayed medical care due to transportation issues were different from the remainder of the US population (Table 1). Demographic groups overrepresented among those facing transportation insecurity included women (62% of adults with transportation insecurity but 52% of adults without transportation insecurity), adults without a partner (63% of adults with transportation insecurity but 41% of adults without transportation insecurity), adults with less education than a bachelor’s degree (79% of adults with transportation insecurity but 54% of adults without transportation insecurity), racial and ethnic minoritized groups (51% of adults with transportation insecurity but 36% of adults without transportation insecurity), and adults not employed at a job or business (71% of adults with transportation insecurity but 39% of adults without transportation insecurity). Additionally, the group with transportation insecurity had a median income 130% of the federal poverty level, which was substantially less than the median of the rest of the population at 400% of the federal poverty level. Subgroup analyses for populations eligible for colorectal, breast, and cervical cancer screening broadly showed similar patterns (eTable 2 in Supplement 1).

**Table 1.** Sociodemographic Characteristics of Respondents Reporting Delaying Medical Care Due to Transportation Compared With the Remainder of the US Population

Characteristic	Care was delayed in past year due to transportation, weighted % (unweighted n = 25 417)^a^^,^^b^	
	No (weighted n = 24 543)	Yes (weighted n = 690)
Gender		
Female	52	62
Male	48	38
Age, y		
18-20	9	15
21-25	16	10
26-30	17	18
31-35	17	17
36-40	16	19
41-45	15	15
46-49	12	6
50-54	18	19
55-59	18	19
60-64	18	23
65-69	16	10
70-74	11	14
75-79	8	4
80-84	5	3
≥85	5	7
Marital status		
Married or living with partner	59	37
Divorced or separated	11	27
Never married	23	28
Widowed	6	7
Other	1	2
Educational attainment		
<High school	6	16
High school or GED	17	27
Some college or associate’s degree	31	36
Bachelor’s degree	26	13
Postgraduate degree	20	8
Employment		
Working for pay at a job or business	61	29
Looking for work	3	9
Not working at a job or business and not looking for work	33	60
Other	3	2
Insurance		
Insured	89	88
Uninsured	10	12
Race and ethnicity		
Hispanic	17	15
Non-Hispanic Asian	6	7
Non-Hispanic Black	12	27
Non-Hispanic White	64	49
Non-Hispanic other^c^	1	2
Federal poverty ratio, median (IQR)	4.01 (2.18-6.60)	1.31 (0.78-2.12)

Abbreviation: GED, General Educational Development.

^a^ Unweighted sample size provided for reference; all descriptive statistics calculated using poststratification weighing.

^b^ Data on the exposure variable, transportation insecurity, and on all other covariates (with the exception of income) were available for over 98% of participants; overall missingness for all variables is given in eTable 1 in Supplement 1.

^c^ Includes American Indian or Alaska Native, multiple race, and primary race not releasable.

Exploratory factor analysis of the 4 items related to neighborhood social support revealed a single factor accounting for 76% of the variance. The single-factor solution was confirmed by a scree plot demonstrating inflection at the second factor (eFigures 1 and 2 in Supplement 1). Factor loadings were strongly positive for a single factor (eTable 3A in Supplement 1), and indeterminacy indices supported the factor score approximations (eTable 3B in Supplement 1). Higher factor scores corresponded to respondents that agreed or strongly agreed that they experienced social support within their neighborhoods. Social support also differed across the same range of sociodemographic categories, including marital status, racial and ethnic group, educational attainment, employment, insurance status, and federal poverty ratio across the samples eligible for colorectal, breast, and cervical cancer screening (eTable 4 in Supplement 1).

In a sample of 10 736 eligible adults in the US population, we found that 6978 (65%) were adherent to colorectal cancer screening; of 5823 eligible adults, 4539 (78%) were adherent to breast cancer screening; and of 7932 eligible adults, 6756 (85%) were adherent to cervical cancer screening (Table 2). Examining crude frequencies, adults experiencing either transportation insecurity or low social support received cancer screening less frequently across all cancer types (Table 2). Among adults 50 to 74 years of age eligible for colorectal cancer screening, transportation insecurity was negatively associated with screening in a univariable model (odds ratio [OR], 0.72 [95% CI, 0.54-0.95]) but not when adjusted for sociodemographic characteristics (OR, 0.87 [95% CI, 0.65-1.15]) (Table 3). Among women 50 to 74 years of age, transportation insecurity was negatively associated with breast cancer screening in both univariable (OR, 0.47 [95% CI, 0.32-0.70) and fully adjusted (OR, 0.59 [95% CI, 0.40-0.86]) models. Finally, transportation insecurity was negatively associated with cervical cancer screening in a univariable model (OR, 0.54, [95% CI, 0.36-0.82]) but not in the fully adjusted model (OR, 0.73 [95% CI, 0.46-1.13]).

**Table 2.** Population Adherence to USPSTF Cancer Screening, Stratified by Transportation Insecurity and Social Support

Cancer screening type	Respondents adherent to screening, %^a^				
	Total population	Transportation insecurity		Social support	
		Yes	No	Low	High
Colorectal	65	57	65	61	69
Breast	78	62	77	74	79
Cervical	85	75	85	83	86

Abbreviation: USPSTF, United States Preventive Services Task Force.

^a^ Percentages generated using weighted analysis.

**Table 3.** Association of Transportation Insecurity and of Social Support With Cancer Screening Among Patients Eligible for Breast, Colorectal, or Cervical Cancer Screening

Cancer screening type	Screening parameters	Eligible population (% of 2018 US adults)^a^	Model	OR (95% CI)
Transportation insecurity				
Colorectal	Fecal immunochemical test within 1 y, stool DNA within 3 y, CT colonography within 5 y, flexible sigmoidoscopy within 5 y, colonoscopy within 10 y	Adults ages 50-74 y (37%)	Unadjusted	0.72 (0.54-0.95)
			Adjusted^b^	0.87 (0.65-1.15)
Breast	Mammogram within 2 y	Women ages 50-74 y (19%)	Unadjusted	0.47 (0.32-0.70)
			Adjusted	0.59 (0.40-0.86)
Cervical	Papanicolaou test within 3 y (age, 21-30 y), or within 5 y (age, 30-65 y)	Women ages 21-65 y who have never had a hysterectomy (33%)	Unadjusted	0.54 (0.36-0.82)
			Adjusted	0.73 (0.46-1.13)
Social support				
Colorectal	Fecal immunochemical test within 1 y, stool DNA within 3 y, CT colonography within 5 y, flexible sigmoidoscopy within 5 y, colonoscopy within 10 y	Adults ages 50-74 y (37%)	Unadjusted	1.22 (1.16-1.28)
			Adjusted^b^	1.12 (1.06-1.17)
Breast	Mammogram within 2 y	Women ages 50-74 y (19%)	Unadjusted	1.19 (1.11-1.28)
			Adjusted	1.13 (1.05-1.22)
Cervical	Papanicolaou test within 3 y (age, 21-30 y), or within 5 y (age, 30-65 y)	Women ages 21-65 y who have never had a hysterectomy (33%)	Unadjusted	1.14 (1.06-1.22)
			Adjusted	1.01 (0.93-1.10)

Abbreviations: CT, computed tomography; OR, odds ratio.

^a^ Based on United States Preventive Services Task Force recommendations in 2018.

^b^ Adjusted for gender (colorectal cancer screen), age, race and ethnicity, marital status, percentage of federal poverty level, highest household educational attainment, employment, insurance, and personal cancer history.

A similar strategy was adopted to understand the association between social support and adherence to recommended cancer screening (Table 3). Social support scores were positively associated with colorectal cancer screening in both univariable (OR, 1.22 [95% CI, 1.16-1.28]) and fully adjusted (OR, 1.12 [95% CI, 1.06-1.17]) models. Similarly, breast cancer screening was positively associated with social support in both univariable (OR, 1.19 [95% CI, 1.11-1.28]) and fully adjusted (OR, 1.13 [95% CI, 1.05-1.22]) models. In contrast, social support was negatively associated with cervical cancer screening in a univariable model (OR, 1.14 [95% CI, 1.06-1.22]); however, there was no association between social support and cervical cancer screening in a fully adjusted model (OR, 1.01 [95% CI, 0.93-1.10]).

For each of the 3 cancer screening types examined, there was no significant interaction between transportation insecurity and social support, suggesting absence of a buffering effect (eTable 5 in Supplement 1). Finally, all results were robust to sensitivity analyses and bootstrapping (eTable 6A-C in Supplement 1).

## Discussion

In this cohort study of 25 417 US adults, we found that transportation insecurity and social support—a closely related social determinant of health—were statistically significantly associated with adherence to USPSTF-recommended cancer screening. After adjustment for sociodemographic, economic, and clinical covariates, transportation insecurity was negatively associated with breast cancer screening but was not associated with cervical or colorectal cancer screening in this sample. After full adjustment for confounders, social support also was positively associated with adherence to both colorectal and breast cancer screening but was not associated with cervical cancer screening in this sample. There were no interaction effects between transportation insecurity and social support in this sample.

Geographic distance has been extensively studied as a determinant of health care outcomes, but whether patients are able to travel for health care services has been largely overlooked. One in 4 US residents experiences some transportation insecurity, and 3% of the population is severely affected, which is typologized by frequently skipping trips, being housebound, and transportation issues negatively affecting relationships and emotions.^6^ As 3% of participants reported delaying medical care due to transportation issues, it seems likely that our exposure item dichotomized the study population into the most transportation-insecure and the remainder of the sample, which would include adults experiencing transportation insecurity to a lesser extent. The contrast between the low proportion of US residents in our cohort who reported delaying medical care due to transportation (3%) and the proportion of US residents reporting any transportation insecurity (25%) may reflect prioritization of medical trips over trips for other purposes, such as shopping or socialization. For example, Mexican-American immigrants facing transportation insecurity borrow cars more frequently for medical trips than any other trip type.^14^ Similarly, seniors who have ceased driving curtail social and recreational trips but continue to travel for medical appointments and errands, nearly always traveling as passengers in a private vehicle.^23,24^ However, reliance on others for medical trips strains relationships by placing a burden on friends and family.^25^ While medical trips may continue, it remains unknown if trips for acute and preventative care continue at the same frequency, or whether medical care that is perceived as nonessential is forgone to reduce the burden on others.

We found that transportation insecurity was associated with 41% decreased odds of adherence to breast cancer screening. The association between geographic access to mammography and stage at diagnosis has been studied vis-à-vis travel distance, facility density, and a 2-step floating catchment area, with 1 systematic review reporting heterogeneity and mixed results in the association between geographic accessibility, mammography adherence, and stage at diagnosis.^9^ However, many of these studies omit an essential element: access to transportation. One analysis combining regional health care records and vehicle registration found that women without a vehicle registered to their household had a higher risk of mammography nonadherence, and that the risk of mammography nonadherence increased more rapidly with increasing distance from the screening site among women without access to a vehicle.^12^ Transportation access may be particularly salient for breast cancer screening, as women overall have less access to shared vehicles, and their access is predicated on the presence of young children in the household.^26^ As women age, they are more likely than men to have ceased driving,^27^ and elderly women are more negatively impacted by driving cessation.^28^ Age- and gender-related differences in access to transportation may partially explain the heterogenous association between transportation insecurity and cervical, colorectal, and breast cancer screening in this national sample, though additional data are needed to test this hypothesis.

As social support provides access to transportation,^29^ and losing access to transportation compromises social relationships,^30^ we evaluated whether neighborhood social support was associated with cancer screening adherence. We did not observe a significant interaction effect between transportation insecurity and social support in any model. Similar to several smaller studies,^31,32,33,34,35,36,37^ we did find that a composite measure of social support positively predicted breast and colorectal cancer screening. It is unclear why social support was not associated with cervical cancer screening in this cohort, as social support was found to increase the odds of cervical cancer screening in the only other large US population study to date of which we are aware.^31^

Although Medicaid universally covers nonemergency medical transportation, local implementation is often onerous for users with poor reliability,^38^ and Medicaid expansion is not available in all states. Medicare funding for medically necessary transportation is also inconsistent: commercial Medicare Advantage plans often include a transportation benefit, but Medicare Parts A and B do not. However, local services and philanthropic organizations do provide some stop-gap coverage, particularly for senior citizens and persons with disabilities. Notably, the American Cancer Society Road to Recovery program offers transportation for cancer screening. Our finding that a 41% reduction in the odds of breast cancer screening was associated with severe transportation insecurity suggests clinicians should screen patients at the time of mammography referral to ensure that they have access to transportation, as there may be resources available.

### Strengths and Limitations

The strengths of this study include the large, nationally representative sample, the high survey response rate, and the use of in-person interviewers. Additionally, we used individual-level data on confounders, such as income and educational attainment, which are often aggregated by zip code tabulation areas or census blocks in similar work.

The study also has several limitations that should be noted. First, the NHIS is a cross-sectional survey with predetermined questions. Therefore, we cannot comment on the temporal or causal relationships between our exposure, confounding, and outcome variables, as all variables (unless otherwise noted) represent snapshot values in time and could have changed between the most recent cancer screening and the next interval screening due. However, all cross-sectional studies of health care disparities in cancer screening are subject to this same limitation. Due to the predetermined nature of the questions, there may be residual confounding unaccounted for in our model. The NHIS relies on participant recall for adherence to cancer screening, although this may be ameliorated by the standardized clarification interviewers could offer for participants unsure of the test or procedure in question. In addition, transportation insecurity was measured using a single question, which likely dichotomized the sample into the 2% to 3% of people with severe transportation insecurity^6^ vs the remainder of the population. Similarly, the social support questions in the 2018 NHIS are not specifically mapped to domains of social support (material, emotional, and informational),^16^ and we found that their variation was best explained by a single latent factor. In other studies, both tangible and emotional support items were associated with mammography^32^ and colorectal cancer screening,^36^ suggesting additional nuance to the association between social support and cancer screening. Finally, although neighborhood social support is particularly vital to meeting the health needs of persons living at or near the poverty line,^19^ social support from within the family unit also influences cancer screening behavior, and we were unable to account for familial social support beyond marital status. None of these limitations can be addressed in data collected in prior iterations of the NHIS, but we hope that increased awareness of transportation insecurity and social support as powerful social determinants of health will lead to the inclusion of additional relevant items.

## Conclusions

In this cohort study of US adults eligible for cancer screening, we found that transportation insecurity was associated with reduced odds of breast cancer screening and that social support was associated with increased odds of breast and colorectal cancer screening. Our findings suggest that transportation and social support are relevant to cancer prevention. Continued interrogation of the roles of transportation and social support—understudied social determinants of health—can help to pinpoint where health care payer, research, and philanthropy dollars can be spent to achieve equity in outcomes of the cancer care continuum.

## Notes

Supplement 1. **eTable 1.** Missingness of all variables included in the analysis in the 2018 National Health Interview Survey “sample adult” population **eTable 2A.** Sociodemographic characteristics of respondents eligible for colorectal cancer screening, split by transportation insecurity **eTable 2B.** Sociodemographic characteristics of respondents eligible for breast cancer screening, split by transportation insecurity **eTable 2C.** Sociodemographic characteristics of respondents eligible for cervical cancer screening, split by transportation insecurity **eFigure 1.** Scree plot demonstrating 1-factor solution for exploratory factor analysis of all neighborhood social support variables **eFigure 2.** Distributions of the four neighborhood social support items included in the 2018 NHIS survey **eTable 3A**. Factor loadings **eTable 3B.** Indeterminacy indices and validity coefficients for exploratory factor analysis **eTable 4A**. Sociodemographic characteristics of respondents eligible for colorectal cancer screening, split by median neighborhood social support **eTable 4B.** Sociodemographic characteristics of respondents eligible for breast cancer screening, split by median neighborhood social support **eTable 4C.** Sociodemographic characteristics of respondents eligible for cervical cancer screening, split by median neighborhood social support **eTable 5.** Effects of transportation insecurity and social support on cancer screening, in fully adjusted models including an interaction term for transportation insecurity and social support **eTable 6A.** Sensitivity analyses for effects of transportation security on cancer screening, with confidence intervals achieved via bootstrapping **eTable 6B.** Sensitivity analyses for effects of neighborhood social support on cancer screening, with confidence intervals achieved via bootstrapping **eTable 6C.** Sensitivity analyses for effects of transportation security and social support on cancer screening, adjusted for visit with primary care provider in the past year in addition to all other covariates

Supplement 2.
Data Sharing Statement
