# Supplementary material for: Transportation Insecurity, Social Support, and Adherence to Cancer Screening
Source: JAMA Netw Open. 2025 Jan 30;8(1):e2457336. doi: 10.1001/jamanetworkopen.2024.57336 (PMC11783192; doi:10.1001/jamanetworkopen.2024.57336)
Supplement: Supplement 1. — eTable 1. Missingness of all variables included in the analysis in the 2018 National Health Interview Survey “sample adult” population eTable 2A. Sociodemographic characteristics of respondents eligible for colorectal cancer screening, split by transportation insecurity eTable 2B. Sociodemographic characteristics of respondents eligible for breast cancer screening, split by transportation insecurity eTable 2C. Sociodemographic characteristics of respondents eligible for cervical cancer screening, split by transportation insecurity eFigure 1. Scree plot demonstrating 1-factor solution for exploratory factor analysis of all neighborhood social support variables eFigure 2. Distributions of the four neighborhood social support items included in the 2018 NHIS survey eTable 3A. Factor loadings eTable 3B. Indeterminacy indices and validity coefficients for exploratory factor analysis eTable 4A. Sociodemographic characteristics of respondents eligible for colorectal cancer screening, split by median neighborhood social support eTable 4B. Sociodemographic characteristics of respondents eligible for breast cancer screening, split by median neighborhood social support eTable 4C. Sociodemographic characteristics of respondents eligible for cervical cancer screening, split by median neighborhood social support eTable 5. Effects of transportation insecurity and social support on cancer screening, in fully adjusted models including an interaction term for transportation insecurity and social support eTable 6A. Sensitivity analyses for effects of transportation security on cancer screening, with confidence intervals achieved via bootstrapping eTable 6B. Sensitivity analyses for effects of neighborhood social support on cancer screening, with confidence intervals achieved via bootstrapping eTable 6C. Sensitivity analyses for effects of transportation security and social support on cancer screening, adjusted for visit with primary care provider in the past year in addition to all oth [file jamanetwopen-e2457336-s001.pdf]

Supplementary Online Content

Pohl AL, Aderonmu AA, Grab JD, Cohen-Tigor LA, Morris AM. Transportation insecurity, social support, and adherence to cancer screening. *JAMA Netw Open*. 2025;8(1):e2457336. doi:10.1001/jamanetworkopen.2024.57336

eTable 1. Missingness of all variables included in the analysis in the 2018 National Health Interview Survey "sample adult" population
eTable 2A. Sociodemographic characteristics of respondents eligible for colorectal cancer screening, split by transportation insecurity
eTable 2B. Sociodemographic characteristics of respondents eligible for breast cancer screening, split by transportation insecurity
eTable 2C. Sociodemographic characteristics of respondents eligible for cervical cancer screening, split by transportation insecurity
eFigure 1. Scree plot demonstrating 1-factor solution for exploratory factor analysis of all neighborhood social support variables
eFigure 2. Distributions of the four neighborhood social support items included in the 2018 NHIS survey
eTable 3A. Factor loadings
eTable 3B. Indeterminacy indices and validity coefficients for exploratory factor analysis
eFigure 2. Distributions of the four neighborhood social support items included in the 2018 NHIS survey
eTable 4A. Sociodemographic characteristics of respondents eligible for colorectal cancer screening, split by median neighborhood social support
eTable 4B. Sociodemographic characteristics of respondents eligible for breast cancer screening, split by median neighborhood social support
eTable 4C. Sociodemographic characteristics of respondents eligible for cervical cancer screening, split by median neighborhood social support
eTable 5. Effects of transportation insecurity and social support on cancer screening, in fully adjusted models including an interaction term for transportation insecurity and social support
eTable 6A. Sensitivity analyses for effects of transportation security on cancer screening, with confidence intervals achieved via bootstrapping
eTable 6B. Sensitivity analyses for effects of neighborhood social support on cancer screening, with confidence intervals achieved via bootstrapping
eTable 6C. Sensitivity analyses for effects of transportation security and social support on cancer screening, adjusted for visit with primary care provider in the past year in addition to all other covariates

This supplementary material has been provided by the authors to give readers additional information about their work.

© 2025 Pohl AL et al. *JAMA Network Open*.

eTable 1. Missingness of all variables included in the analysis in the 2018 National Health Interview Survey "sample adult" population

	Overall Missingness (%)	Missingness stratified by transportation insecurity		Missingness stratified by social support	
		No (%)	Yes (%)	High (%)	Low (%)
**Exposure Variables**					
Transportation Insecurity	0.8				
Social Support	1.7				
**Covariates**					
Gender	0	-	-	-	-
Age	0	-	-	-	-
Marital Status	0.2	0.2	0.0	0.1	0.2
Education	0.2	0.2	0.1	0.1	0.2
Employment	<0.1	<0.1	0.0	<0.1	<0.1
Insurance	0.5	0.5	0.0	0.3	0.6
Race/Ethnicity	0	-	-	-	-
Federal Poverty Ratio	16	16	18	15	16
Personal Hx Colorectal Cancer[1]	0.2	0.2	0.0	<0.1	0.4
Personal Hx Breast Cancer[2]	0.1	0.1	0.0	<0.1	0.2
Peronals Hx Cervical Cancer[3]	<0.1	<0.1	0.0	<0.1	0.1
**Outcome Variables**					
Colorectal Cancer Screening[1,4]	0	-	-	-	-
Breast Cancer Screening[2,4]	0	-	-	-	-
Cervical Cancer Screening[3,4]	0	-	-	-	-

[1]Calcuated within population eligible for colorectal cancer screening. [2] Calculated within population eligible for breast cancer screening. [3] Calculated within population eligible for cervical cancer screening. [4] If a participant eligible for cancer screening answered "Don't know" or did not answer questions about cancer screening, they were assigned to the "non-adherent" outcome group.

© 2025 Pohl AL et al. *JAMA Network Open*.

eTable 2A. Sociodemographic characteristics of respondents eligible for colorectal cancer screening, split by transportation insecurity

Ever delay care in past year due to transportation?<br>unweighted *n* = 10,736	No<br>(weighted %)	Yes<br>(weighted %)
Gender		
Female	52	59
Male	48	41
Age (by groups)		
50-54	22	21
55-59	22	23
60-64	23	23
65-69	19	16
70-74	14	17
Marital status		
Married/Living with partner	68	37
Divorced/Separated	16	36
Never married	8	14
Widowed	6	10
Unknown/Other	1	3
Education		
Less than High School	6	18
High School/GED	18	25
Some college/Associate's Degree	31	38
Bachelor's Degree	25	13
Postgraduate Degree	20	6
Employment		
Working for pay at a job or business	52	15
Looking for work	2	5
Not working at a job or business and not looking for work	42	77
Insurance		
Insured	94	91
Uninsured	6	9
Race/Ethnicity		
Hispanic	12	15
Non-Hispanic White	71	52
Non-Hispanic Asian	5	4
Non-Hispanic Black	11	27
Non-Hispanic Other	1	3
Personal history of colorectal cancer		
No	99	98
Yes	1	2
Federal Poverty Ratio		
Median	4.01	1.31
Interquartile Range	2.18 – 6.60	0.78 – 2.12

© 2025 Pohl AL et al. *JAMA Network Open*.

eTable 2B. Sociodemographic characteristics of respondents eligible for breast cancer screening, split by transportation insecurity

Ever delay care in past year due to transportation? (unweighted *n* = 5,823)	No (weighted %)	Yes (weighted %)
Age (by groups)		
50-54	22	21
55-59	22	21
60-64	23	22
65-69	19	19
70-74	15	17
Marital Status		
Married/Living with partner	64	34
Divorced/Separated	19	37
Never married	7	11
Widowed	9	15
Other	1	2
Education		
Less than High School	6	16
High School/GED	18	24
Some college/Associate's Degree	32	40
Bachelor's Degree	24	16
Postgraduate Degree	20	4
Employment		
Working for pay at a job or business	47	15
Looking for work	2	6
Not working at a job or business and not looking for work	47	77
Other	4	2
Insurance		
Insured	94	94
Uninsured	6	6
Race/Ethnicity		
Hispanic	12	14
Non-Hispanic White	70	54
Non-Hispanic Asian	5	5
Non-Hispanic Black	11	24
Non-Hispanic Other	1	2
Personal history of breast cancer		
No	94	93
Yes	6	7
Federal Poverty Ratio		
Median	3.87	1.15
Interquartile Range	2.08 – 6.45	0.71 – 1.93

© 2025 Pohl AL et al. *JAMA Network Open*.

eTable 2C. Sociodemographic characteristics of respondents eligible for cervical cancer screening, split by transportation insecurity

Ever delay care in past year due to transportation? (unweighted *n* = 7,932)	No (weighted %)	Yes (weighted %)
Age (by groups)		
21-34	37	35
35-49	33	30
50-65	30	35
Marital Status		
Married/Living with partner	62	44
Divorced/Separated	11	29
Never married	23	23
Widowed	2	3
Other	1	1
Education		
Less than High School	5	10
High School/GED	15	31
Some college/Associate's Degree	30	31
Bachelor's Degree	28	21
Postgraduate Degree	22	8
Employment		
Working for pay at a job or business	69	34
Looking for work	3	15
Not working at a job or business and not looking for work	24	48
Other	4	3
Insurance		
Insured	89	84
Uninsured	11	16
Race/Ethnicity		
Hispanic	19	21
Non-Hispanic White	59	47
Non-Hispanic Asian	8	7
Non-Hispanic Black	13	20
Non-Hispanic Other	1	4
Personal history of cervical cancer		
No	99	98
Yes	1	2
Federal Poverty Ratio		
Median	3.51	1.15
Interquartile Range	1.80 – 5.95	0.69 – 2.14

© 2025 Pohl AL et al. *JAMA Network Open*.

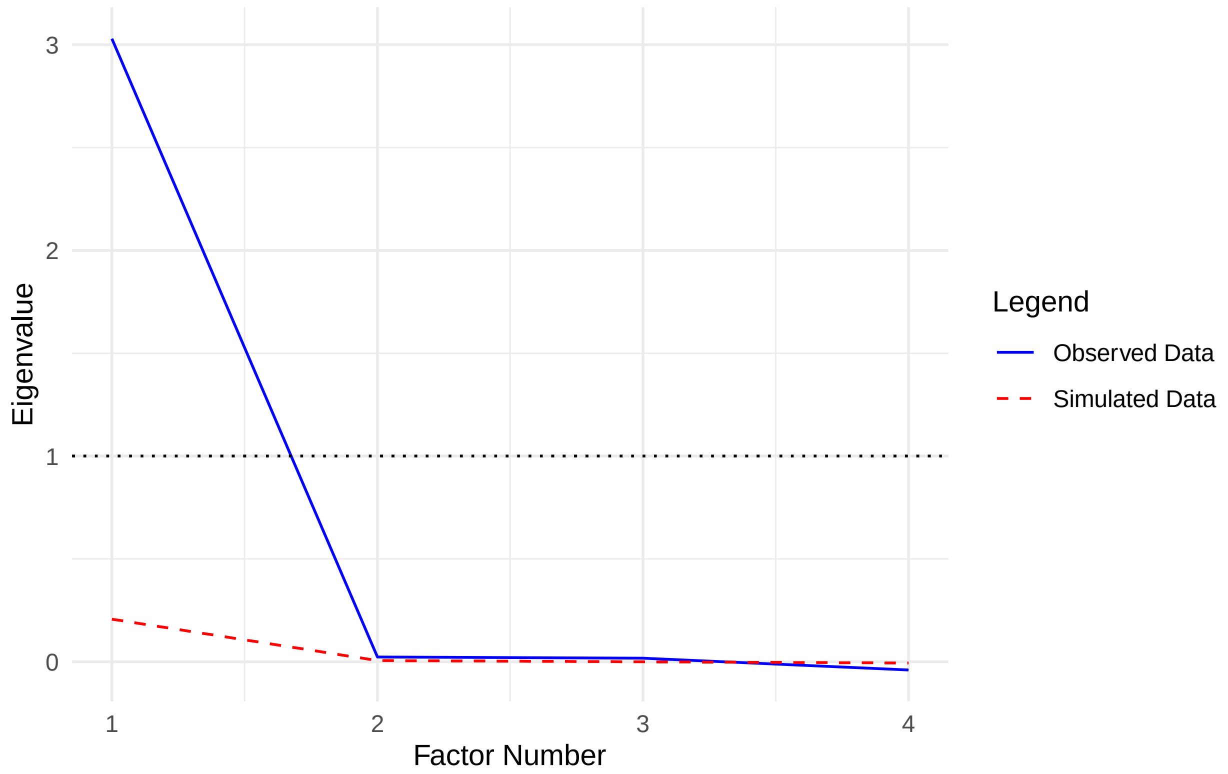

eFigure 1. Scree plot demonstrating 1-factor solution for exploratory factor analysis of all neighborhood social support variables. A single-factor solution is also accurate by Kaiser's criteria (retain factors with Eigenvalues > 1). Parallel analysis (simulated data) would suggest a 3-factor solution, but the difference between Eigenvalues for the observed and simulated data for factors 2 & 3 is negligible, again suggesting a 1-factor solution.

© 2025 Pohl AL et al. *JAMA Network Open*.

eTable 3A. Factor loadings

Item	Factor loading [95%CI]
There are people I can count on in this neighborhood	0.90 [0.90, 0.90]
People in this neighborhood help each other out	0.90 [0.90, 0.90]
This is a close-knit neighborhood	0.83 [0.82, 0.83]
People in this neighborhood can be trusted	0.85 [0.84, 0.85]

eTable 3B. Indeterminacy indices and validity coefficients for exploratory factor analysis

Measure[1]	Guideline[2]	Factor 1
Indeterminacy Indices		
Correlation of (regression) scores with factors	0 – 1, high values desirable	0.96
Multiple R square of scores with factors	0 – 1, high values desirable	0.93
Minimum correlation of possible factor scores	-1 – 1, high positive values desirable	0.86
Validity coefficient	-1 – 1, high positive values desirable, should exceed 0.80	0.96

[1]Grice's final two measures for evaluating factor score approximations, univocality and correlational accuracy, cannot be calculated from a single factor solution as they are based on factor score matrices. [2]Guidelines for interpretation provided from Grice 2001.

© 2025 Pohl AL et al. *JAMA Network Open*.

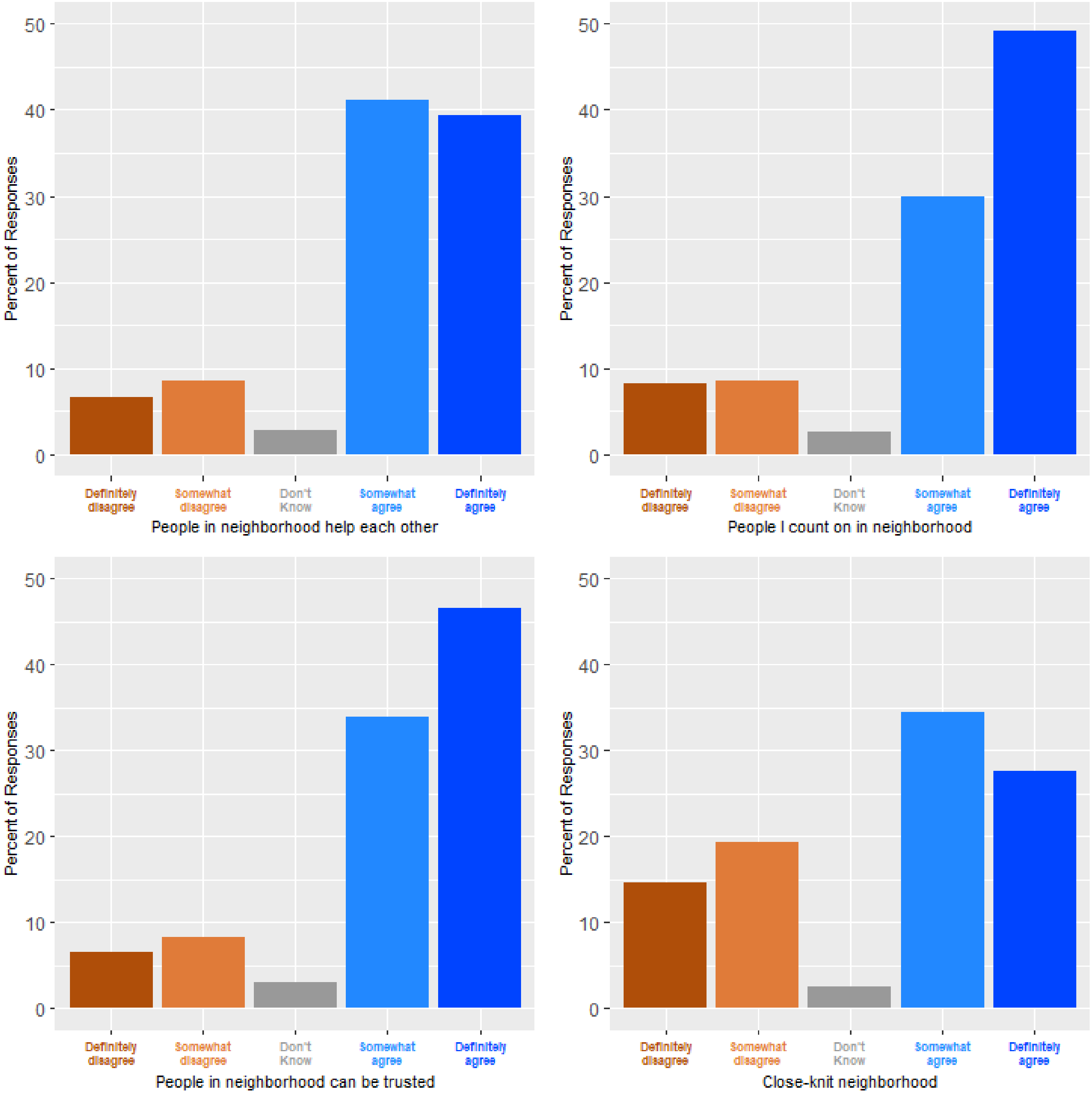

eFigure 2. Distributions of the four neighborhood social support items included in the 2018 NHIS survey. Most Americans somewhat or definitely agree that people in their neighborhood help each other out (81.7%), that there are people they can count on in their neighborhood (80.6%), that people in their neighborhood can be trusted (81.8%), and a weaker majority believes that they live in a close-knit neighborhood (63.0%).

© 2025 Pohl AL et al. *JAMA Network Open*.

eTable 4A. Sociodemographic characteristics of respondents eligible for colorectal cancer screening, split by median neighborhood social support

Neighborhood Social Support (Unweighted *n* = 10,736)	Low (weighted %)	High (weighted %)
Gender		
Female	51	53
Male	49	47
Age (by groups)		
50-54	24	21
55-59	23	21
60-64	22	24
65-69	18	19
70-74	14	15
Marital status		
Married/Living with partner	62	72
Divorced/Separated	20	14
Never married	10	7
Widowed	7	6
Unknown/Other	2	1
Education		
Less than High School	8	5
High School/GED	19	17
Some college/Associate's Degree	33	30
Bachelor's Degree	23	26
Postgraduate Degree	17	22
Employment		
Working for pay at a job or business	51	51
Looking for work	3	2
Not working at a job or business and not looking for work	44	43
Insurance		
Insured	92	95
Uninsured	8	5
Race/Ethnicity		
Hispanic	15	9
Non-Hispanic White	63	77
Non-Hispanic Asian	6	5
Non-Hispanic Black	14	9
Non-Hispanic Other	2	1
Personal history of colorectal cancer		
No	99	99
Yes	1	1
Federal Poverty Ratio		
Median	3.32	4.48
Interquartile Range	1.71 – 5.78	2.45 – 7.23

© 2025 Pohl AL et al. *JAMA Network Open*.

eTable 4B. Sociodemographic characteristics of respondents eligible for breast cancer screening, split by median neighborhood social support

Neighborhood Social Support (unweighted *n* = 5,823)	Low (weighted %)	High (weighted %)
Age (by groups)		
50-54	23	21
55-59	23	21
60-64	22	23
65-69	19	18
70-74	13	16
Marital Status		
Married/Living with partner	58	68
Divorced/Separated	22	16
Never married	10	5
Widowed	9	9
Other	1	1
Education		
Less than High School	8	5
High School/GED	19	17
Some college/Associate's Degree	34	31
Bachelor's Degree	22	26
Postgraduate Degree	17	22
Employment		
Working for pay at a job or business	46	46
Looking for work	3	1
Not working at a job or business and not looking for work	49	48
Other	3	4
Insurance		
Insured	93	96
Uninsured	7	4
Race/Ethnicity		
Hispanic	16	9
Non-Hispanic White	62	76
Non-Hispanic Asian	6	5
Non-Hispanic Black	14	10
Non-Hispanic Other	1	1
Personal history of breast cancer		
No	95	94
Yes	5	6
Federal Poverty Ratio		
Median	3.13	432
Interquartile Range	1.58 – 5.42	2.37 – 7.10

© 2025 Pohl AL et al. *JAMA Network Open*.

eTable 4C. Sociodemographic characteristics of respondents eligible for cervical cancer screening, split by median neighborhood social support

Neighborhood Social Support (unweighted *n* = 7,932)	Low (weighted %)	High (weighted %)
Age (by groups)		
21-34	42	30
35-49	32	34
50-65	26	35
Marital Status		
Married/Living with partner	56	69
Divorced/Separated	12	10
Never married	28	18
Widowed	2	2
Other	2	1
Education		
Less than High School	6	4
High School/GED	18	13
Some college/Associate's Degree	34	27
Bachelor's Degree	26	30
Postgraduate Degree	17	27
Employment		
Working for pay at a job or business	68	67
Looking for work	4	2
Not working at a job or business and not looking for work	24	26
Other	3	4
Insurance		
Insured	87	91
Uninsured	13	9
Race/Ethnicity		
Hispanic	24	14
Non-Hispanic White	50	69
Non-Hispanic Asian	8	7
Non-Hispanic Black	17	9
Non-Hispanic Other	1	1
Personal history of cervical cancer		
No	100	99
Yes	0	1
% of Federal Poverty Level		
Median	2.82	4.30
Interquartile Range	1.43 – 4.82	2.31 – 6.92

© 2025 Pohl AL et al. *JAMA Network Open*.

eTable 5. Effects of transportation insecurity and social support on cancer screening, in fully adjusted models including an interaction term for transportation insecurity and social support

Cancer Screening	Eligible Population (% of 2018 US adults)	Model	OR [95% CI]
**Colorectal**: FIT within 1 year, stool-DNA within 3 years, CT colonography within 5 years, flex sig within 5 years, colonoscopy within 10 years	Adults 50-74 (37%)	Transportation Insecurity	0.89 [0.66 – 1.20]
		**Social Support**	**1.12 [1.06 – 1.18]**
		Interaction	0.94 [0.76 – 1.16]
**Breast:** Mammogram within 2 years	Women 50-74 (19%)	**Transportation Insecurity**	**0.60 [0.40 – 0.89]**
		**Social Support**	**1.13 [1.04 – 1.22]**
		Interaction	0.93 [0.68 – 1.28]
**Cervical:** Pap within 3 years (21-30), or within 5 years (30-65)	Women 21-65 who have never had a hysterectomy (33%)	Transportation Insecurity	0.70 [0.44 – 1.10]
		Social Support	1.01 [0.93 – 1.10]
		Interaction	0.90 [0.65 – 1.25]

© 2025 Pohl AL et al. *JAMA Network Open*.

eTable 6A. Sensitivity analyses for effects of transportation security on cancer screening, with confidence intervals achieved via bootstrapping

**Cancer Screening**	**Eligible Population** (% of 2018 US adults)	**Model**	**Transportation Insecurity** OR [95% CI]
**Colorectal**: FIT within 1 year, stool-DNA within 3 years, CT colonography within 5 years, flex sig within 5 years, colonoscopy within 10 years	Adults 50-74 (37%)	**Unadjusted**	**0.72 [0.55 – 0.94]**
		Adjusted	0.87 [0.66 – 1.15]
**Breast:** Mammogram within 2 years	Women 50-74 (19%)	**Unadjusted**	**0.47 [0.32 – 0.70]**
		**Adjusted**	**0.59 [0.39 – 0.89]**
**Cervical:** Pap within 3 years (21-30), or within 5 years (30-65)	Women 21-65 who have never had a hysterectomy (33%)	**Unadjusted**	**0.54 [0.36 – 0.80]**
		Adjusted	0.72 [0.46 – 1.13]

eTable 6B. Sensitivity analyses for effects of neighborhood social support on cancer screening, with confidence intervals achieved via bootstrapping

**Cancer Screening**	**Eligible Population** (% of 2018 US adults)	**Model**	**Social Support** OR [95% CI]
Colorectal: FIT within 1 year, stool-DNA within 3 years, CT colonography within 5 years, flex sig within 5 years, colonoscopy within 10 years	Adults 50-74 (37%)	**Unadjusted**	**1.22 [1.16 – 1.28]**
		**Adjusted**	**1.12 [1.06 – 1.18]**
Breast: Mammogram within 2 years	Women 50-74 (19%)	**Unadjusted**	**1.19 [1.11 – 1.29]**
		**Adjusted**	**1.13 [1.05 – 1.22]**
Cervical: Pap within 3 years (21-30), or within 5 years (30-65)	Women 21-65 who have never had a hysterectomy (33%)	**Unadjusted**	**1.14[1.06 – 1.22]**
		Adjusted	1.01 [0.93 – 1.10]

© 2025 Pohl AL et al. *JAMA Network Open*.

eTable 6C. Sensitivity analyses for effects of transportation security and social support on cancer screening, adjusted for visit with primary care provider in the past year in addition to all other covariates

**Cancer Screening**	**Eligible Population** (% of 2018 US adults)	**Model**	**OR [95% CI]**
**Colorectal**: FIT within 1 year, stool-DNA within 3 years, CT colonography within 5 years, flex sig within 5 years, colonoscopy within 10 years	Adults 50-74 (37%)	Transportation Insecurity	0.84 [0.62 – 1.13]
		**Social Support**	**1.13 [1.07 – 1.20]**
**Breast:** Mammogram within 2 years	Women 50-74 (19%)	**Transportation Insecurity**	**0.58 [0.39 – 0.85]**
		**Social Support**	**1.15 [1.05 – 1.25]**
**Cervical:** Pap within 3 years (21-30), or within 5 years (30-65)	Women 21-65 who have never had a hysterectomy (33%)	Transportation Insecurity	0.69 [0.44 – 1.08]
		Social Support	1.01 [0.93 – 1.11]

This is a sensitivity analysis which includes a covariate which asked if participants had seen or talked to a general doctor who treats a variety of illnesses (a doctor in general practice, family medicine, or internal medicine) during the past 12 months

© 2025 Pohl AL et al. *JAMA Network Open*.
