# Supplementary material for: Transportation Insecurity, Social Support, and Adherence to Cancer Screening
Source: JAMA Netw Open. 2025 Jan 30;8(1):e2457336. doi: 10.1001/jamanetworkopen.2024.57336 (PMC11783192; doi:10.1001/jamanetworkopen.2024.57336)
Supplement: Supplement 2. — Data Sharing Statement [file jamanetwopen-e2457336-s002.pdf]

Data Sharing Statement

Pohl. Transportation Insecurity, Social Support, and Adherence to Cancer Screening. *JAMA Netw Open*. Published January 30, 2025. doi:10.1001/jamanetworkopen.2024.57336

Data
Data available: No

Additional Information
Explanation for why data not available: This is publicly available data.
